# Supplementary material for: Socialization of Providencia stuartii Enables Resistance to Environmental Insults
Source: Microorganisms. 2022 Apr 25;10(5):901. doi: 10.3390/microorganisms10050901 (PMC9144925; doi:10.3390/microorganisms10050901)
Supplement: Supplementary file 1 [file microorganisms-10-00901-s001.zip › microorganisms-1678166-supplementary.pdf]

Supplementary Information

Socialization of *Providencia stuartii* enables resistance to environmental insults.

Authors:

Julie Lopes[1], Guillaume Tetreau[1], Kevin Pounot[1], Mariam El Khatib[1], Jacques-Philippe Colletier[1]

Affiliations:

[1] Univ. Grenoble Alpes – CEA – CNRS, Institut de Biologie Structurale (IBS), Grenoble, France

Correspondence: colletier@ibs.fr

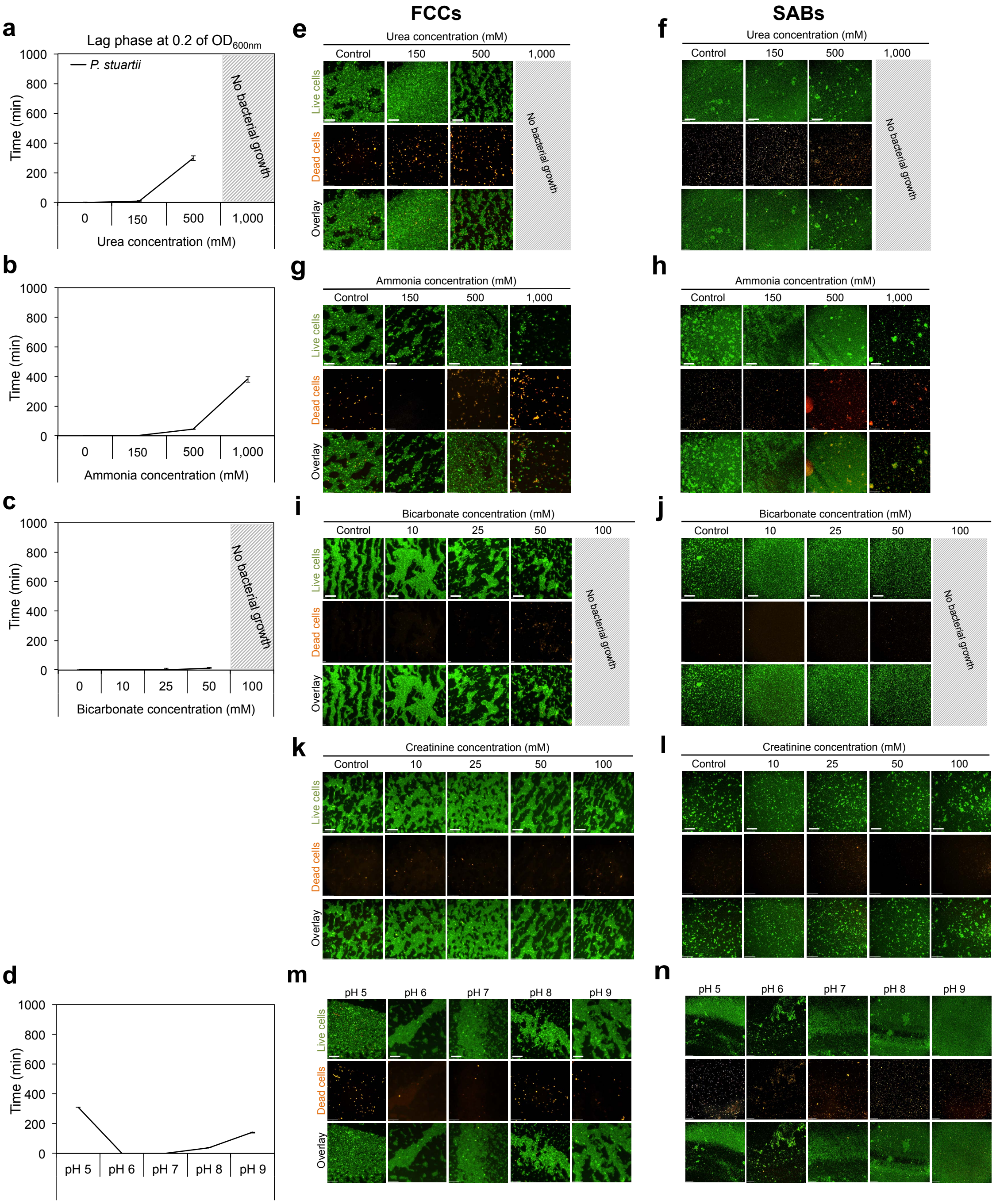

Supplementary Figure S1. *P. stuartii* is highly resistant to catabolites present in the urinary tract. (a-d) The overall impact of environmental cues on *P. stuartii* growth was monitored by determining, at increasing concentrations of the cues or pH, the lag time before reaching an optical density of 0.2 at 600 nm. Panels a, b, c, and d show results for urea, ammonia, bicarbonate and for different pH, respectively. All measurements were performed in triplicates; errors bars outline standard deviations from the average. Grey-hatched rectangles indicate concentrations at which bacteria were unable to grow. (**e-n**) Epifluorescence microscopy was used to monitor FCC (e,g,I,k,m) and SAB formation (f,h,j,l,n) in the presence of urea (e,f), ammonium (g,h), bicarbonate (i,j), creatinine (k,l) and at various pH (m,n). All bacteria are labelled by the permeant DNA stain Syto9 (green channel), but only dead cells are labelled by propidium iodide (red channel). For each condition, an overlay of the two channels is shown. Grey-hatched rectangles indicate concentrations at which bacteria were unable to grow. Scale bars correspond to 50 and 200 µm in FCC and SAB micrographs, respectively.

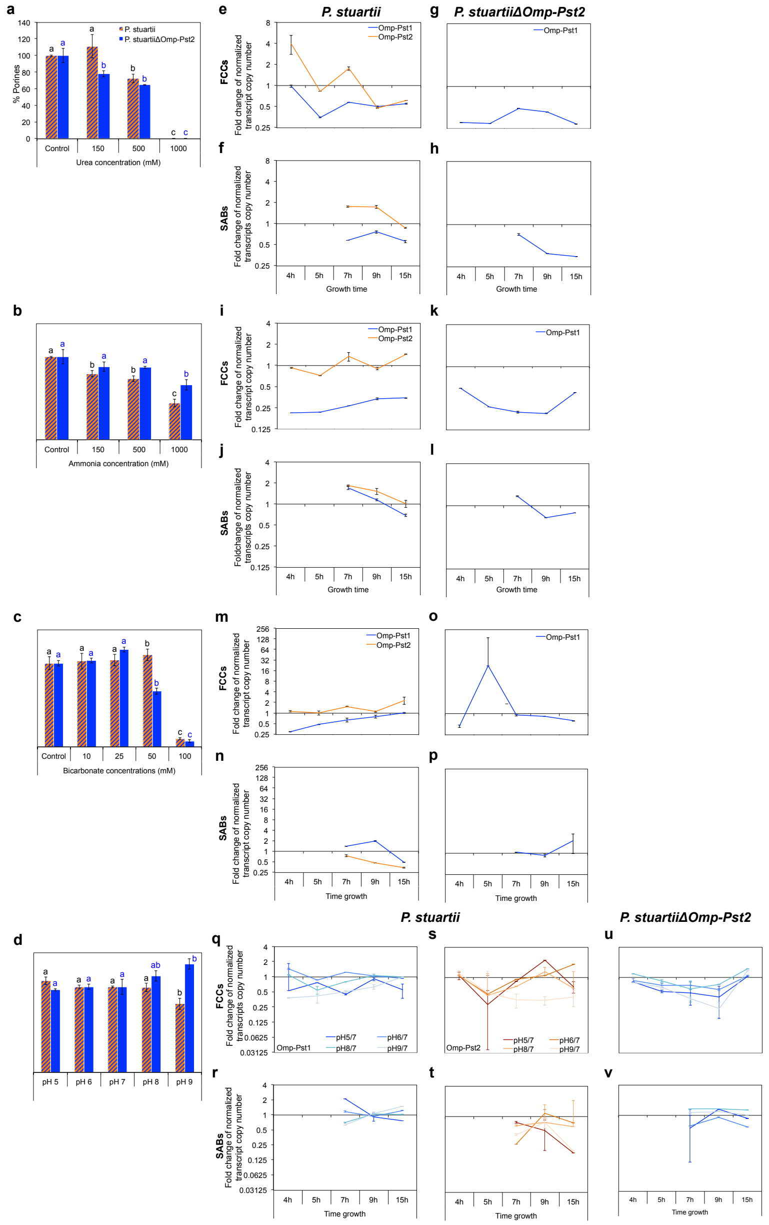

Supplementary Figure S2. Regulation of porin expression in the presence of environmental cues is more pronounced in FCC than SAB, and sometimes opposed. (a-d) Porin abundance in the OM of *P. stuartii* FCC and SAB cells grown in presence of increasing concentrations of urea (panel a), ammonia (b), bicarbonate (c) and to various pH (d) was evaluated by image processing of digitized SDS-PAGE gels using ImageJ. Plots show percent increase or decrease in porin abundance in the OM, after normalization of intensity counts from porin bands of exposed bacteria by those of unexposed bacteria. All measurements were performed in triplicates; errors bars outline standard deviations from the average. Different letters above the bars indicate significant differences ($p < 0.05$; ANOVA followed by post-hoc Tukey HSD test; see statistical indicators in Supplementary Table S1)**. (e-v)** Immediate changes in the expression of Omp-Pst1 (blue) and Omp-Pst2 (orange) in WT *P. stuartii* or *P. stuartii*ΔOmp-Pst2 FCCs and SABs cells were monitored by RT-qPCR following 30 min incubation with 500 mM of urea (panels e, f and g, h, respectively), 500 mM ammonia (i, j and k, l), to 50 mM bicarbonate (m, n and o, p), or to pH variation (q, r, s, t and u, v). For each growth time point, we report the ratio of normalized transcript copy numbers between cells exposed and not exposed (control) to the cues. All measurements were performed in triplicates; errors bars outline standard deviations from the average. Different letters above the bars indicate significant differences ($p < 0.05$; ANOVA followed by post-hoc Tukey HSD test; see statistical indicators in Supplementary Table S2)**.**

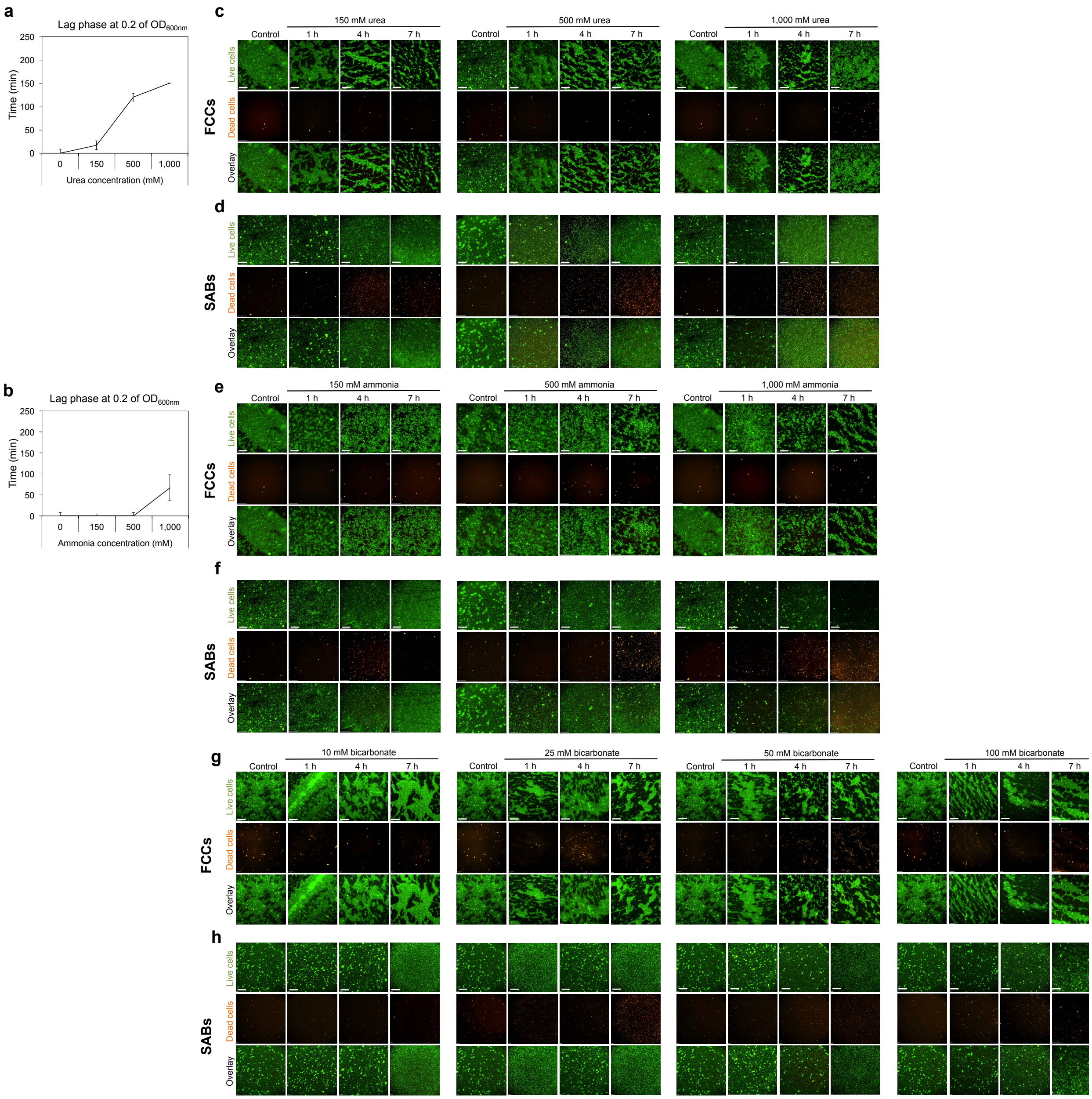

Supplementary Figure S3. FCC and SAB cells are generally more resistant than their plantktonic counterparts. (a-b) Recovery of preformed *P. stuartii* FCC and SAB cells after sudden exposure to urea (a) or ammonia (b) was monitored by determining, at increasing concentrations of these, the lag time before reaching an optical density of 0.2 at 600 nm. All measurements were performed in triplicates; errors bars outline standard deviations from the average. (**e-h**) Epifluorescence microscopy was used to monitor FCC (e,g,I,k,m) and SAB survival and recovery (f,h,j,l,n) in presence of increasing concentrations of urea (panels c and d, respectively), ammonia (e, f), and bicarbonate (g, h). All bacteria are labelled by the permeant DNA stain Syto9 (green channel), but only dead cells are labelled by propidium iodide (red channel). For each condition, an overlay of the two channels is shown. Grey-hatched rectangles indicate concentrations at which bacteria were unable to grow. Scale bars correspond to 50 and 200 µm in FCC and SAB micrographs, respectively. Note that preformed *P. stuartii* FCC and SAB generally resist better to the cues than their developing counterparts, as illustrated by survival of FCC and SAB at 1 M urea and 100 mM bicarbonate, where new cells do no grow (Supplementary Fig. 1). An exception is ammonium, which disrupts SAB formed in its absence although preformed FCC survive and new FCC and SAB can form (Supplementary Fig. 1).

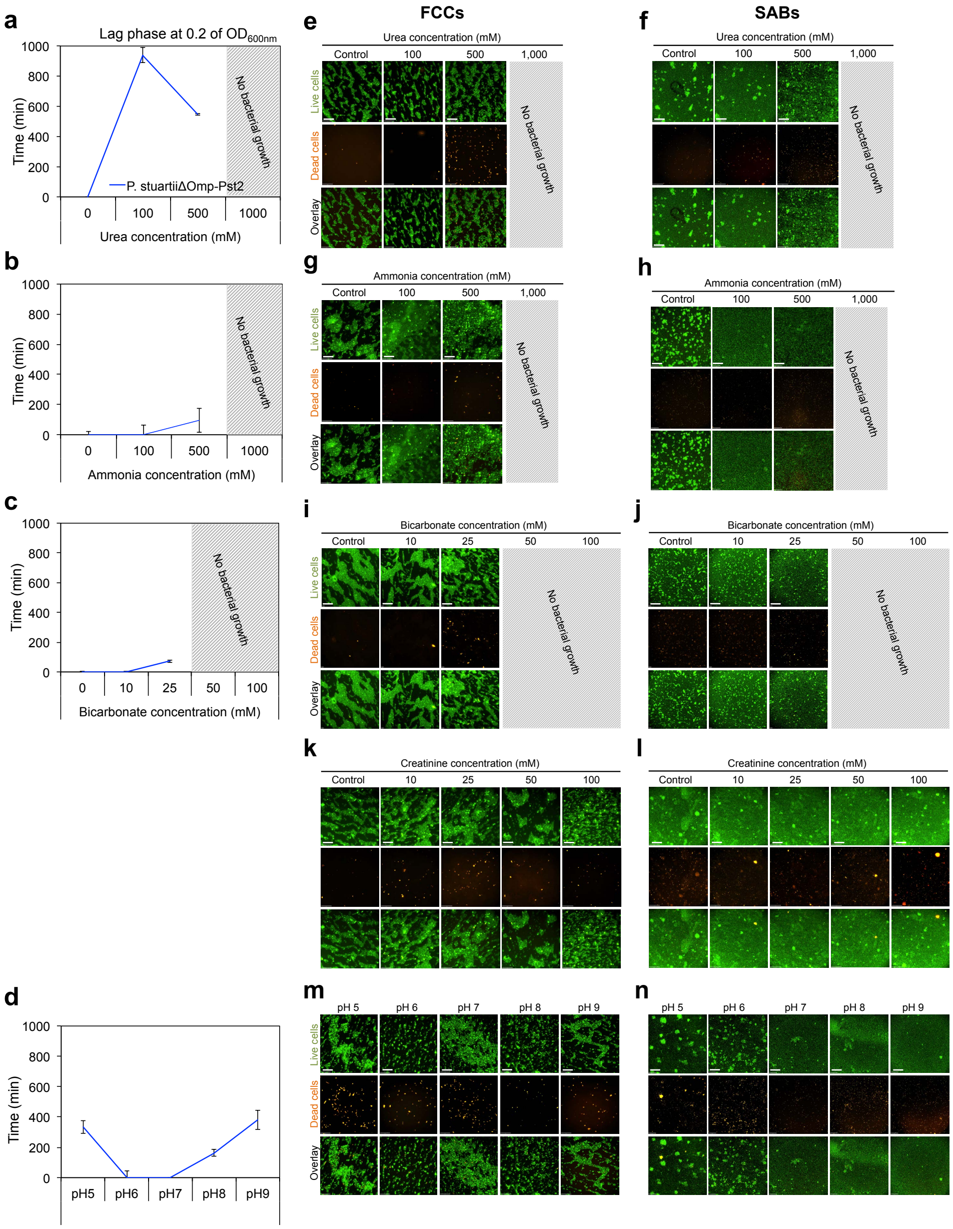

Supplementary Figure S4. Omp-Pst2 benefits resistance of *P. stuartii* to its pathophysiological environment. (a-d) The contribution of Omp-Pst2 in adapting to high concentration of urea (a), ammonia (b), bicarbonate (c) or to different pHs (d) was evaluated on the *P. stuartii*ΔOmp-Pst2 strain by determining, at increasing concentrations of the cues or pH, the lag time before reaching an optical density of 0.2 at 600 nm, and comparing results to the wild-type (Supplementary Fig. 1). All measurements were performed in triplicates; errors bars outline standard deviations from the average. Grey-hatched rectangles indicate concentrations at which bacteria were unable to grow. (**e-n**) Epifluorescence microscopy was used to monitor FCC (e,g,I,k,m) and SAB formation (f,h,j,l,n) by *P. stuartii*ΔOmp-Pst2 cells in the presence of urea (e,f), ammonium (g,h), bicarbonate (i,j), creatinine (k,l) and at various pH (m,n). All bacteria are labelled by the permeant DNA stain Syto9 (green channel), but only dead cells are labelled by propidium iodide (red channel). For each condition, an overlay of the two channels is shown. Grey-hatched rectangles indicate concentrations at which bacteria were unable to grow. Scale bars correspond to 50 and 200 µm in FCC and SAB micrographs, respectively.

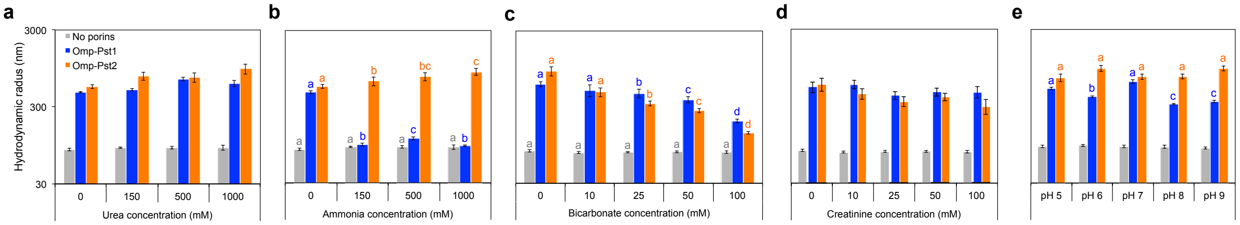

Supplementary Figure S5. Omp-Pst1 and Omp-Pst2 self-association into DOT can be challenged by environmental cues. LDAO-solubilized Omp-Pst1 (blue) and Omp-Pst2 (orange) were reconstituted in ~50-nm radius LUV, in presence of increasing concentrations of urea (a), ammonia (b), bicarbonate (c), creatinine (d) or at various pH (e), and after 24 h incubation with biobeads, the hydrodynamic radius of proteoliposomes was measured by DLS. The hydrodynamic radii of LUVs incubated at with the cues but without porin were also measured (grey). Different letters above the bars indicate significant differences ($p < 0.05$; ANOVA followed by post-hoc Tukey HSD test; see statistical indicators in Supplementary Table S3).

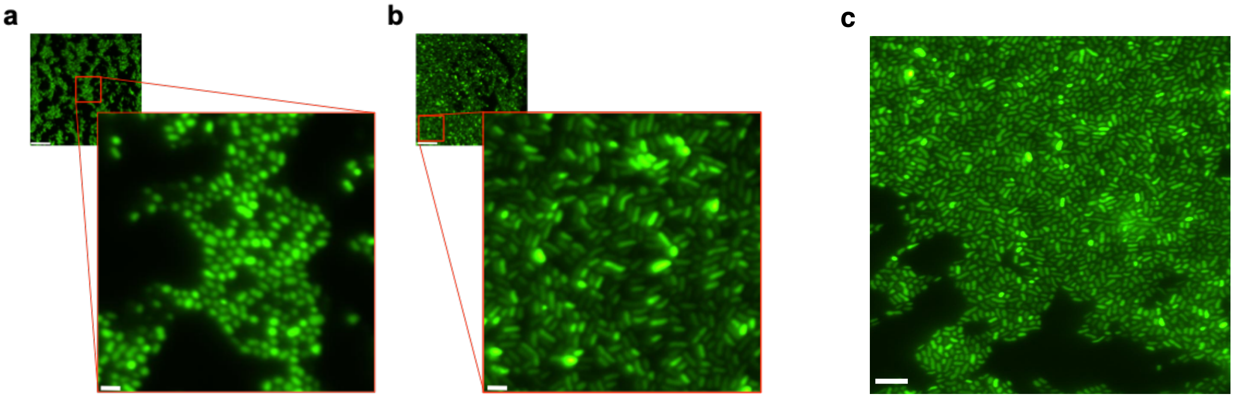

Supplementary Figure S6. The orientation of *P. stuartii* cells differs in native FCC and in FCC formed in presence of environmental cues. Close-up views of FCCs developed in the presence of urea (a) and ammonia (b), showing “standing” and “seated” orientations of cells, respectively. (c) Unexposed FCC feature cells in the standing orientation. Epifluorescence microscopy was used to image FCC cells post-labelling by the permeant DNA stain Syto9 (green channel). The scale bars in the small and large panels are 50 μm and 5 μm, respectively.

Supplementary Table S1 – Statistical analysis from SDS-PAGE gels experiments on porin abundance in OMs. All experiments were conducted for at least three biologically independent replicates. Technical replicates were averaged to produce replicate means that were subsequently used for analysis. Mean values were compared within and between groups using one-way ANOVA followed by Tukey's post hoc for two-two comparisons. Differences were considered statistically significant if p<0.05 (True).

GLM –SDS-PAGE Gels

All statistic parameters – *P. stuartii*:

```
                 Generalized Linear Model Regression Results
==============================================================================
Dep. Variable:                      Y   No. Observations:                   39
Model:                            GLM   Df Residuals:                       35
Model Family:                   Gamma   Df Model:                            3
Link Function:          inverse_power   Scale:                         0.20020
Method:                          IRLS   Log-Likelihood:                    inf
Date:                Thu, 14 Feb 2019   Deviance:                       212.99
Time:                        16:59:38   Pearson chi2:                     7.01
No. Iterations:                     8   Covariance Type:             nonrobust
====================================================================================
                       coef    std err          z      P>|z|      [0.025      0.975]
------------------------------------------------------------------------------------
Intercept            0.4455      0.395      1.129      0.259      -0.328       1.219
Urea              4.854e-05   1.21e-05      4.011      0.000    2.48e-05    7.23e-05
Ammonium          1.704e-05   6.75e-06      2.524      0.012    3.81e-06    3.03e-05
Urea:Ammonium     8.351e-16   7.63e-16      1.094      0.274   -6.61e-16    2.33e-15
pH                   0.0362      0.057      0.638      0.523      -0.075       0.147
Urea:pH              0.0003   8.47e-05      4.011      0.000       0.000       0.001
Ammonium:pH          0.0001   4.73e-05      2.524      0.012    2.67e-05       0.000
Urea:Ammonium:pH          0          0        nan        nan           0           0
====================================================================================
```

Bicarbonate – *P. stuartii*:

```
                 Generalized Linear Model Regression Results
==============================================================================
Dep. Variable:                      Y   No. Observations:                   15
Model:                            GLM   Df Residuals:                       13
Model Family:                   Gamma   Df Model:                            1
Link Function:          inverse_power   Scale:                         0.29338
Method:                          IRLS   Log-Likelihood:                -136.43
Date:                Mon, 20 May 2019   Deviance:                       5.1540
Time:                        09:29:53   Pearson chi2:                     3.81
No. Iterations:                     7   Covariance Type:             nonrobust
=================================================================================
                    coef    std err          z      P>|z|      [0.025      0.975]
---------------------------------------------------------------------------------
Intercept         0.0001   3.41e-05      4.046      0.000    7.11e-05       0.000
Concentration  3.294e-06   1.26e-06      2.606      0.009    8.17e-07    5.77e-06
=================================================================================
```

All statistic parameters – *P. stuartii*ΔOmp-Pst2:

```
                 Generalized Linear Model Regression Results
==============================================================================
Dep. Variable:                 Values   No. Observations:                   39
Model:                            GLM   Df Residuals:                       35
Model Family:                   Gamma   Df Model:                            3
Link Function:          inverse_power   Scale:                         0.19051
Method:                          IRLS   Log-Likelihood:                    inf
Date:                Mon, 20 May 2019   Deviance:                       212.67
Time:                        09:37:42   Pearson chi2:                     6.67
No. Iterations:                     8   Covariance Type:             nonrobust
==============================================================================
                 coef    std err          z      P>|z|      [0.025      0.975]
------------------------------------------------------------------------------
Intercept      1.2797      0.498      2.572      0.010       0.305       2.255
Urea           0.0041      0.001      4.534      0.000       0.002       0.006
Ammonia        0.0005      0.000      1.670      0.095   -9.18e-05       0.001
pH            -0.0586      0.069     -0.855      0.392      -0.193       0.076
==============================================================================
```

Bicarbonate – *P. stuartii*ΔOmp-Pst2:

```
                 Generalized Linear Model Regression Results
==============================================================================
```

```
Dep. Variable:                      Y   No. Observations:                   15
Model:                            GLM   Df Residuals:                       13
Model Family:                   Gamma   Df Model:                            1
Link Function:          inverse_power   Scale:                         0.31456
Method:                          IRLS   Log-Likelihood:                -133.53
Date:                Mon, 20 May 2019   Deviance:                       7.3202
Time:                        09:30:46   Pearson chi2:                     4.09
No. Iterations:                     7   Covariance Type:             nonrobust
=================================================================================
                    coef    std err          z      P>|z|      [0.025      0.975]
---------------------------------------------------------------------------------
Intercept         0.0001   3.93e-05      3.569      0.000    6.33e-05       0.000
Concentration  7.247e-06   2.08e-06      3.491      0.000    3.18e-06    1.13e-05
=================================================================================
```

2-2 comparison (Tukey) – *P. stuartii*

Urea condition:

```
Multiple Comparison of Means - Tukey HSD,FWER=0.05
=============================================
group1 group2 meandiff  lower   upper  reject
---------------------------------------------
  0     150    0.1542  -0.1792  0.4876 False
  0     500   -0.3759  -0.7093 -0.0425  True
  0     1000  -1.3651  -1.6985 -1.0317  True
 150    500   -0.5301  -0.8635 -0.1966  True
 150    1000  -1.5193  -1.8527 -1.1858  True
 500    1000  -0.9892  -1.3226 -0.6558  True
```

Ammonia condition:

```
Multiple Comparison of Means - Tukey HSD,FWER=0.05
=============================================
group1 group2 meandiff  lower   upper  reject
---------------------------------------------
  0     150   -0.2778  -0.4343 -0.1212  True
  0     500   -0.3618  -0.5183 -0.2052  True
  0     1000  -0.7592  -0.9157 -0.6026  True
 150    500    -0.084  -0.2405  0.0726 False
 150    1000  -0.4814  -0.6379 -0.3248  True
 500    1000  -0.3974  -0.5539 -0.2408  True
```

Bicarbonate condition:

```
  Multiple Comparison of Means - Tukey HSD,FWER=0.05
=====================================================
group1 group2  meandiff    lower      upper    reject
-----------------------------------------------------
  0      10    975.8383   211.0188  1740.6579   True
  0      25    756.3667   -8.4529   1521.1862  False
  0      50    780.3097   15.4901   1545.1292   True
  0     100   -4409.2103 -5174.0299 -3644.3908  True
  10     25   -219.4717  -984.2912   545.3479  False
  10     50   -195.5287  -960.3482   569.2909  False
  10    100   -5385.0487 -6149.8682 -4620.2291  True
  25     50     23.943   -740.8765   788.7625  False
  25    100   -5165.577  -5930.3965 -4400.7575  True
  50    100    -5189.52  -5954.3395 -4424.7005  True
```

pH condition:

```
Multiple Comparison of Means - Tukey HSD,FWER=0.05
=============================================
group1 group2 meandiff  lower   upper  reject
---------------------------------------------
  5      6    -0.0942  -0.2838  0.0954 False
  5      7    -0.0976  -0.2871  0.092  False
  5      8    -0.1051  -0.2947  0.0844 False
  5      9    -0.3684   -0.558 -0.1788  True
  6      7    -0.0034  -0.1929  0.1862 False
  6      8    -0.0109  -0.2005  0.1786 False
  6      9    -0.2742  -0.4638 -0.0846  True
  7      8    -0.0076  -0.1972  0.182  False
```

7	9	-0.2708	-0.4604	-0.0813	True
8	9	-0.2632	-0.4528	-0.0737	True

2-2 comparison (Tukey) – *P. stuartii*ΔOmp-Pst2

Urea condition:

Multiple Comparison of Means - Tukey HSD,FWER=0.05

group1	group2	meandiff	lower	upper	reject
0	150	-0.2351	-0.4253	-0.045	True
0	500	-0.3753	-0.5654	-0.1851	True
0	1000	-1.0683	-1.2585	-0.8782	True
150	500	-0.1401	-0.373	0.0928	False
150	1000	-0.8332	-1.0661	-0.6003	True
500	1000	-0.6931	-0.926	-0.4602	True

Ammonia condition:

Multiple Comparison of Means - Tukey HSD,FWER=0.05

group1	group2	meandiff	lower	upper	reject
0	150	-0.1283	-0.3409	0.0842	False
0	500	-0.135	-0.3475	0.0775	False
0	1000	-0.3613	-0.5738	-0.1487	True
150	500	-0.0066	-0.2669	0.2536	False
150	1000	-0.2329	-0.4932	0.0274	False
500	1000	-0.2263	-0.4865	0.034	False

Bicarbonate condition:

Multiple Comparison of Means - Tukey HSD,FWER=0.05

group1	group2	meandiff	lower	upper	reject
0	10	346.731	-289.3837	982.8457	False
0	25	154.9093	-481.2053	791.024	False
0	50	-2002.9833	-2639.098	-1366.8687	True
0	100	-4633.1367	-5269.2513	-3997.022	True
10	25	-191.8217	-827.9363	444.293	False
10	50	-2349.7143	-2985.829	-1713.5997	True
10	100	-4979.8677	-5615.9823	-4343.753	True
25	50	-2157.8927	-2794.0073	-1521.778	True
25	100	-4788.046	-5424.1607	-4151.9313	True
50	100	-2630.1533	-3266.268	-1994.0387	True

pH condition:

Multiple Comparison of Means - Tukey HSD,FWER=0.05

group1	group2	meandiff	lower	upper	reject
5	6	0.0374	-0.2237	0.2985	False
5	7	0.0388	-0.1744	0.252	False
5	8	0.1749	-0.0862	0.436	False
5	9	0.3246	0.0635	0.5857	True
6	7	0.0014	-0.2118	0.2146	False
6	8	0.1375	-0.1236	0.3986	False
6	9	0.2872	0.0261	0.5483	True
7	8	0.1361	-0.0771	0.3493	False
7	9	0.2858	0.0726	0.499	True
8	9	0.1497	-0.1114	0.4108	False

Supplementary Table S2 – Statistical analysis from RT-qPCR experiments on porin expression into FCCs and SABs. All experiments were conducted for at least three biologically independent replicates. Technical replicates were averaged to produce replicate means that were subsequently used for analysis. Mean values were compared within and between groups using one-way ANOVA followed by Tukey's post hoc for two-two comparisons. Differences were considered statistically significant if $p<0.05$ (True).

GLM – RTqPCR – *P. stuartii*

Parameter correlations: Urea - Omp-Pst1

```
                 Generalized Linear Model Regression Results
==============================================================================
Dep. Variable:                      Y   No. Observations:                   88
Model:                            GLM   Df Residuals:                       80
Model Family:                   Gamma   Df Model:                            7
Link Function:          inverse_power   Scale:                         0.22000
Method:                          IRLS   Log-Likelihood:                -1032.9
Date:                Tue, 21 May 2019   Deviance:                       19.302
Time:                        15:24:28   Pearson chi2:                     17.6
No. Iterations:                     7   Covariance Type:             nonrobust
========================================================================================================
                                           coef    std err          z      P>|z|      [0.025      0.975]
--------------------------------------------------------------------------------------------------------
Intercept                             1.976e-05   4.34e-06      4.553      0.000    1.13e-05    2.83e-05
C(Phenotype)[T.Floating]             -5.015e-06   4.73e-06     -1.060      0.289   -1.43e-05    4.26e-06
Time                                 -5.654e-07   3.72e-07     -1.518      0.129    -1.3e-06    1.64e-07
C(Phenotype)[T.Floating]:Time         1.242e-07   4.13e-07      0.301      0.763   -6.85e-07    9.33e-07
Urea                                 -8.492e-09   3.04e-08     -0.279      0.780   -6.81e-08    5.11e-08
C(Phenotype)[T.Floating]:Urea         1.565e-08   3.27e-08      0.478      0.632   -4.85e-08    7.98e-08
Time:Urea                             3.323e-09   3.17e-09      1.049      0.294   -2.89e-09    9.53e-09
C(Phenotype)[T.Floating]:Time:Urea -1.773e-09   3.47e-09     -0.511      0.609   -8.57e-09    5.03e-09
========================================================================================================
```

Parameter correlations: Urea - Omp-Pst2

```
                 Generalized Linear Model Regression Results
==============================================================================
Dep. Variable:                      Y   No. Observations:                   88
Model:                            GLM   Df Residuals:                       80
Model Family:                   Gamma   Df Model:                            7
Link Function:          inverse_power   Scale:                         0.78223
Method:                          IRLS   Log-Likelihood:                -881.32
Date:                Tue, 21 May 2019   Deviance:                       56.367
Time:                        15:25:17   Pearson chi2:                     62.6
No. Iterations:                     8   Covariance Type:             nonrobust
========================================================================================================
                                           coef    std err          z      P>|z|      [0.025      0.975]
--------------------------------------------------------------------------------------------------------
Intercept                                0.0001   4.72e-05      2.651      0.008    3.26e-05       0.000
C(Phenotype)[T.Floating]              8.957e-05   7.12e-05      1.257      0.209      -5e-05       0.000
Time                                  -4.41e-06   3.91e-06     -1.127      0.260   -1.21e-05    3.26e-06
C(Phenotype)[T.Floating]:Time        -1.412e-06   6.45e-06     -0.219      0.827     -1.4e-05    1.12e-05
Urea                                 -1.312e-07   1.32e-07     -0.995      0.320     -3.9e-07    1.27e-07
C(Phenotype)[T.Floating]:Urea        -9.149e-08   2.12e-07     -0.431      0.666   -5.08e-07    3.25e-07
Time:Urea                              6.08e-09   1.15e-08      0.530      0.596   -1.64e-08    2.86e-08
C(Phenotype)[T.Floating]:Time:Urea  1.064e-08   2.18e-08      0.488      0.625   -3.21e-08    5.33e-08
========================================================================================================
```

Parameter correlations: Ammonia - Omp-Pst1

```
                 Generalized Linear Model Regression Results
==============================================================================
Dep. Variable:                      Y   No. Observations:                   89
Model:                            GLM   Df Residuals:                       81
Model Family:                   Gamma   Df Model:                            7
Link Function:          inverse_power   Scale:                         0.18900
Method:                          IRLS   Log-Likelihood:                -1039.4
Date:                Tue, 21 May 2019   Deviance:                       15.403
Time:                        15:26:44   Pearson chi2:                     15.3
No. Iterations:                     7   Covariance Type:             nonrobust
============================================================================================================
                                               coef    std err          z      P>|z|      [0.025      0.975]
------------------------------------------------------------------------------------------------------------
Intercept                                 1.976e-05   4.02e-06      4.913      0.000    1.19e-05    2.76e-05
C(Phenotype)[T.Floating]                 -5.015e-06   4.39e-06     -1.143      0.253   -1.36e-05    3.58e-06
Time                                     -5.654e-07   3.45e-07     -1.638      0.101   -1.24e-06    1.11e-07
C(Phenotype)[T.Floating]:Time             1.242e-07   3.82e-07      0.325      0.745   -6.25e-07    8.74e-07
```

```
Ammonium                                  -2.697e-08   1.19e-08     -2.259      0.024   -5.04e-08   -3.57e-09
C(Phenotype)[T.Floating]:Ammonium          8.771e-08   2.01e-08      4.364      0.000    4.83e-08    1.27e-07
Time:Ammonium                               1.78e-09   1.11e-09      1.601      0.109   -3.99e-10    3.96e-09
C(Phenotype)[T.Floating]:Time:Ammonium -4.559e-09     1.8e-09     -2.530      0.011   -8.09e-09   -1.03e-09
==============================================================================================================
```

Parameter correlations: Ammonia - Omp-Pst2

```
                 Generalized Linear Model Regression Results
==============================================================================
Dep. Variable:                      Y   No. Observations:                   89
Model:                            GLM   Df Residuals:                       81
Model Family:                   Gamma   Df Model:                            7
Link Function:          inverse_power   Scale:                         0.74485
Method:                          IRLS   Log-Likelihood:                -869.64
Date:                Tue, 21 May 2019   Deviance:                       51.910
Time:                        15:27:33   Pearson chi2:                     60.3
No. Iterations:                     8   Covariance Type:             nonrobust
==============================================================================================================
                                             coef    std err          z      P>|z|      [0.025      0.975]
--------------------------------------------------------------------------------------------------------------
Intercept                                  0.0001   4.61e-05      2.717      0.007    3.49e-05       0.000
C(Phenotype)[T.Floating]                8.957e-05   6.95e-05      1.289      0.198   -4.67e-05       0.000
Time                                    -4.41e-06   3.82e-06     -1.155      0.248   -1.19e-05    3.07e-06
C(Phenotype)[T.Floating]:Time          -1.412e-06   6.29e-06     -0.225      0.822   -1.37e-05    1.09e-05
Ammonium                                1.805e-07    2.8e-07      0.644      0.520   -3.69e-07     7.3e-07
C(Phenotype)[T.Floating]:Ammonium       4.068e-07   4.24e-07      0.961      0.337   -4.23e-07    1.24e-06
Time:Ammonium                          -5.772e-09    2.3e-08     -0.251      0.802   -5.08e-08    3.93e-08
C(Phenotype)[T.Floating]:Time:Ammonium -3.723e-08   3.37e-08     -1.106      0.269   -1.03e-07    2.87e-08
==============================================================================================================
```

Parameter correlations: Bicarbonate - Omp-Pst1

```
                 Generalized Linear Model Regression Results
==============================================================================
Dep. Variable:                 Values   No. Observations:                   48
Model:                            GLM   Df Residuals:                       40
Model Family:                   Gamma   Df Model:                            7
Link Function:          inverse_power   Scale:                0.28273516624
Method:                          IRLS   Log-Likelihood:                -618.86
Date:                Fri, 05 Jul 2019   Deviance:                       12.855
Time:                        13:51:13   Pearson chi2:                     11.3
No. Iterations:                     8
====================================================================================================
                                              coef    std err          z      P>|z|      [95.0% Conf. Int.]
----------------------------------------------------------------------------------------------------
Intercept                                 1.779e-05    4.1e-06      4.341      0.000     9.76e-06  2.58e-05
C(Phenotypes)[T.Floating]                -1.118e-05   4.31e-06     -2.591      0.010    -1.96e-05 -2.72e-06
Time                                     -1.128e-06   2.75e-07     -4.098      0.000    -1.67e-06 -5.88e-07
C(Phenotypes)[T.Floating]:Time             8.29e-07   2.97e-07      2.791      0.005     2.47e-07  1.41e-06
Cconditions                              -1.554e-08   9.83e-09     -1.581      0.114    -3.48e-08  3.73e-09
C(Phenotypes)[T.Floating]:Cconditions     3.568e-08   1.16e-08      3.063      0.002     1.29e-08  5.85e-08
Time:Cconditions                          1.162e-09   6.73e-10      1.728      0.084    -1.56e-10  2.48e-09
C(Phenotypes)[T.Floating]:Time:Cconditions -2.541e-09 8.11e-10     -3.133      0.002    -4.13e-09 -9.51e-10
====================================================================================================
```

Parameter correlations: Bicarbonate - Omp-Pst2

```
                 Generalized Linear Model Regression Results
==============================================================================
Dep. Variable:                 Values   No. Observations:                   48
Model:                            GLM   Df Residuals:                       40
Model Family:                   Gamma   Df Model:                            7
Link Function:          inverse_power   Scale:               0.350373625544
Method:                          IRLS   Log-Likelihood:                -516.90
Date:                Fri, 05 Jul 2019   Deviance:                       15.804
Time:                        14:02:51   Pearson chi2:                     14.0
No. Iterations:                     7
====================================================================================================
                                              coef    std err          z      P>|z|      [95.0% Conf. Int.]
----------------------------------------------------------------------------------------------------
Intercept                                 2.502e-05   2.08e-05      1.204      0.229    -1.57e-05  6.58e-05
C(Phenotypes)[T.Floating]                -3.345e-05   2.65e-05     -1.262      0.207    -8.54e-05  1.85e-05
Time                                      7.925e-07   2.01e-06      0.394      0.694    -3.15e-06  4.74e-06
C(Phenotypes)[T.Floating]:Time             7.57e-06   3.53e-06      2.143      0.032     6.48e-07  1.45e-05
Cconditions                              -8.522e-08   9.79e-08     -0.870      0.384    -2.77e-07  1.07e-07
C(Phenotypes)[T.Floating]:Cconditions     1.389e-07   1.06e-07      1.312      0.190    -6.87e-08  3.46e-07
Time:Cconditions                          1.602e-08    1.1e-08      1.452      0.146     -5.6e-09  3.76e-08
C(Phenotypes)[T.Floating]:Time:Cconditions -2.859e-08 1.29e-08     -2.221      0.026    -5.38e-08 -3.36e-09
====================================================================================================
```

Parameter correlations: pH - Omp-Pst1

```
                 Generalized Linear Model Regression Results
==============================================================================
Dep. Variable:                      Y   No. Observations:                  156
Model:                            GLM   Df Residuals:                      148
Model Family:                   Gamma   Df Model:                            7
Link Function:          inverse_power   Scale:                         0.29369
Method:                          IRLS   Log-Likelihood:                -1843.2
Date:                Tue, 21 May 2019   Deviance:                       47.729
Time:                        15:29:23   Pearson chi2:                     43.5
No. Iterations:                     7   Covariance Type:             nonrobust
====================================================================================================
                                       coef    std err          z      P>|z|      [0.025      0.975]
----------------------------------------------------------------------------------------------------
Intercept                        -3.249e-05   2.38e-05     -1.365      0.172   -7.91e-05    1.42e-05
C(Phenotype)[T.Floating]          3.936e-05   2.59e-05      1.522      0.128   -1.13e-05    9.01e-05
Time                              3.894e-06   2.45e-06      1.593      0.111   -8.98e-07    8.69e-06
C(Phenotype)[T.Floating]:Time    -3.441e-06   2.61e-06     -1.317      0.188   -8.56e-06    1.68e-06
pH                                7.667e-06   3.44e-06      2.227      0.026     9.2e-07    1.44e-05
C(Phenotype)[T.Floating]:pH      -5.688e-06   3.74e-06     -1.523      0.128    -1.3e-05    1.63e-06
Time:pH                          -6.133e-07   3.43e-07     -1.786      0.074   -1.29e-06    5.97e-08
C(Phenotype)[T.Floating]:Time:pH   4.35e-07   3.67e-07      1.186      0.236   -2.84e-07    1.15e-06
====================================================================================================
```

Parameter correlations: pH - Omp-Pst2

```
                 Generalized Linear Model Regression Results
==============================================================================
Dep. Variable:                      Y   No. Observations:                  155
Model:                            GLM   Df Residuals:                      147
Model Family:                   Gamma   Df Model:                            7
Link Function:          inverse_power   Scale:                         0.90306
Method:                          IRLS   Log-Likelihood:                -1510.8
Date:                Tue, 21 May 2019   Deviance:                       112.64
Time:                        15:28:36   Pearson chi2:                     133.
No. Iterations:                     8   Covariance Type:             nonrobust
====================================================================================================
                                       coef    std err          z      P>|z|      [0.025      0.975]
----------------------------------------------------------------------------------------------------
Intercept                         6.154e-05      0.000      0.222      0.825      -0.000       0.001
C(Phenotype)[T.Floating]             0.0002      0.000      0.511      0.609      -0.001       0.001
Time                              2.285e-06   2.74e-05      0.083      0.934   -5.14e-05     5.6e-05
C(Phenotype)[T.Floating]:Time    -2.104e-05   3.64e-05     -0.578      0.563   -9.23e-05    5.03e-05
pH                                  1.1e-05   3.96e-05      0.278      0.781   -6.65e-05    8.85e-05
C(Phenotype)[T.Floating]:pH      -1.724e-05   5.17e-05     -0.334      0.739      -0.000     8.4e-05
Time:pH                          -7.931e-07   3.88e-06     -0.204      0.838    -8.4e-06    6.82e-06
C(Phenotype)[T.Floating]:Time:pH  3.284e-06   5.26e-06      0.624      0.532   -7.02e-06    1.36e-05
====================================================================================================
```

GLM – RTqPCR – *P. stuartii*ΔOmp-Pst2

Parameter correlations: Urea

```
                 Generalized Linear Model Regression Results
==============================================================================
Dep. Variable:                      Y   No. Observations:                   96
Model:                            GLM   Df Residuals:                       88
Model Family:                   Gamma   Df Model:                            7
Link Function:          inverse_power   Scale:                         0.20053
Method:                          IRLS   Log-Likelihood:                -1144.3
Date:                Tue, 21 May 2019   Deviance:                       19.798
Time:                        16:17:17   Pearson chi2:                     17.6
No. Iterations:                     8   Covariance Type:             nonrobust
======================================================================================================
                                         coef    std err          z      P>|z|      [0.025      0.975]
------------------------------------------------------------------------------------------------------
Intercept                           5.906e-06   2.35e-06      2.509      0.012    1.29e-06    1.05e-05
C(Phenotype)[T.Floating]              1.85e-06   2.72e-06      0.681      0.496   -3.48e-06    7.18e-06
Time                                  2.43e-07   2.34e-07      1.038      0.299   -2.16e-07    7.02e-07
C(Phenotype)[T.Floating]:Time       -7.038e-08   2.84e-07     -0.248      0.804   -6.26e-07    4.86e-07
Urea                                 -5.05e-08   2.39e-08     -2.115      0.034   -9.73e-08    -3.7e-09
C(Phenotype)[T.Floating]:Urea        6.131e-08   2.77e-08      2.215      0.027    7.06e-09    1.16e-07
Time:Urea                            8.384e-09   2.96e-09      2.835      0.005    2.59e-09    1.42e-08
C(Phenotype)[T.Floating]:Time:Urea -5.163e-09   3.54e-09     -1.459      0.144   -1.21e-08    1.77e-09
======================================================================================================
```

Parameter correlations: Ammonia

```
                 Generalized Linear Model Regression Results
==============================================================================
Dep. Variable:                      Y   No. Observations:                   96
Model:                            GLM   Df Residuals:                       88
Model Family:                   Gamma   Df Model:                            7
Link Function:          inverse_power   Scale:                         0.15986
Method:                          IRLS   Log-Likelihood:                -1145.4
Date:                Tue, 21 May 2019   Deviance:                       15.809
Time:                        16:18:11   Pearson chi2:                     14.1
No. Iterations:                     7   Covariance Type:             nonrobust
===================================================================================================
                                          coef    std err          z      P>|z|      [0.025      0.975]
---------------------------------------------------------------------------------------------------
Intercept                            5.906e-06    2.1e-06      2.810      0.005    1.79e-06       1e-05
C(Phenotype)[T.Floating]              1.85e-06   2.43e-06      0.763      0.446   -2.91e-06    6.61e-06
Time                                  2.43e-07   2.09e-07      1.162      0.245   -1.67e-07    6.53e-07
C(Phenotype)[T.Floating]:Time       -7.038e-08   2.53e-07     -0.278      0.781   -5.67e-07    4.26e-07
Ammonium                            -7.175e-09   7.82e-09     -0.918      0.359   -2.25e-08    8.14e-09
C(Phenotype)[T.Floating]:Ammonium    5.693e-08   1.47e-08      3.863      0.000     2.8e-08    8.58e-08
Time:Ammonium                        5.944e-10   8.13e-10      0.731      0.465   -9.98e-10    2.19e-09
C(Phenotype)[T.Floating]:Time:Ammonium -2.649e-09   1.48e-09     -1.785      0.074   -5.56e-09    2.59e-10
===================================================================================================
```

Parameter correlations: Bicarbonate

```
                 Generalized Linear Model Regression Results
==============================================================================
Dep. Variable:                 Values   No. Observations:                   48
Model:                            GLM   Df Residuals:                       40
Model Family:                   Gamma   Df Model:                            7
Link Function:          inverse_power   Scale:                   1.13895772977
Method:                          IRLS   Log-Likelihood:                -612.73
Date:                Fri, 05 Jul 2019   Deviance:                       30.400
Time:                        15:15:31   Pearson chi2:                     45.6
No. Iterations:                     9
==============================================================================================
                                           coef    std err          z      P>|z|      [95.0% Conf. Int.]
----------------------------------------------------------------------------------------------
Intercept                             3.441e-05   1.61e-05      2.135      0.033      2.83e-06   6.6e-05
C(Phenotypes)[T.Floating]            -2.833e-06   1.91e-05     -0.148      0.882     -4.03e-05  3.46e-05
Time                                 -2.157e-06   1.09e-06     -1.988      0.047     -4.28e-06    -3e-08
C(Phenotypes)[T.Floating]:Time        1.842e-07   1.29e-06      0.143      0.886     -2.34e-06  2.71e-06
Cconditions                           8.339e-09   4.73e-08      0.176      0.860     -8.43e-08  1.01e-07
C(Phenotypes)[T.Floating]:Cconditions -5.474e-08  5.21e-08     -1.051      0.293     -1.57e-07  4.74e-08
Time:Cconditions                     -7.075e-10   3.17e-09     -0.223      0.823     -6.92e-09  5.51e-09
C(Phenotypes)[T.Floating]:Time:Cconditions  4.06e-09  3.53e-09  1.149      0.250     -2.86e-09   1.1e-08
==============================================================================================
```

Parameter correlations: pH

```
                 Generalized Linear Model Regression Results
==============================================================================
Dep. Variable:                      Y   No. Observations:                  168
Model:                            GLM   Df Residuals:                      160
Model Family:                   Gamma   Df Model:                            7
Link Function:          inverse_power   Scale:                         0.21959
Method:                          IRLS   Log-Likelihood:                -2016.9
Date:                Tue, 21 May 2019   Deviance:                       34.902
Time:                        16:19:02   Pearson chi2:                     35.1
No. Iterations:                     7   Covariance Type:             nonrobust
=============================================================================================
                                    coef    std err          z      P>|z|      [0.025      0.975]
---------------------------------------------------------------------------------------------
Intercept                      1.953e-05   1.05e-05      1.856      0.063    -1.1e-06    4.02e-05
C(Phenotype)[T.Floating]      -9.239e-06   1.27e-05     -0.725      0.468   -3.42e-05    1.57e-05
Time                          -2.693e-07   1.01e-06     -0.266      0.790   -2.25e-06    1.71e-06
C(Phenotype)[T.Floating]:Time   5.56e-07   1.27e-06      0.436      0.663   -1.94e-06    3.05e-06
pH                            -1.726e-06   1.41e-06     -1.222      0.222   -4.49e-06    1.04e-06
C(Phenotype)[T.Floating]:pH    2.052e-06   1.73e-06      1.183      0.237   -1.35e-06    5.45e-06
Time:pH                        6.412e-08   1.37e-07      0.469      0.639   -2.04e-07    3.32e-07
C(Phenotype)[T.Floating]:Time:pH -1.241e-07   1.74e-07     -0.712      0.476   -4.66e-07    2.17e-07
=============================================================================================
```

2-2 comparison (Tukey) - Urea condition – *P. stuartii*

Omp-Pst1 porin – FCCs

```
        Multiple Comparison of Means - Tukey HSD,FWER=0.05
```

	group1	group2	meandiff	lower	upper	reject
4h	0	500	16574.1498	-30452.3034	63600.6031	False
5h	0	500	-38902.0202	-69962.3523	-7841.6881	True
7h	0	500	-71189.8961	-123737.1743	-18642.618	True
9h	0	500	-63057.0912	-127634.1388	1519.9565	True
15h	0	500	-63286.2712	-114749.5456	-11822.9968	True

Omp-Pst2 porin – FCCs

Multiple Comparison of Means - Tukey HSD,FWER=0.05

	group1	group2	meandiff	lower	upper	reject
4h	0	500	6261.4946	1996.197	10526.7923	True
5h	0	500	-2134.6555	- 12996.94	8727.629	False
7h	0	500	7521.8879	1598.1456	13445.6302	True
9h	0	500	-657.2522	-10717.0073	9402.5029	False
15h	0	500	-539.4299	-9813.6084	8734.7485	False

2-2 comparison (Tukey) - Urea condition – *P. stuartii* – According Time

Omp-Pst1 porin – FCCs

Multiple Comparison of Means - Tukey HSD,FWER=0.05

group1	group2	meandiff	lower	upper	reject
4	5	-69510.7473	-90915.2466	-48106.248	True
4	7	-30288.1114	-51692.6107	-8883.6121	True
4	9	-47009.2685	-68413.7677	-25604.7692	True
4	15	-35263.6879	-56668.1872	-13859.1886	True
5	7	39222.6359	17818.1367	60627.1352	True
5	9	22501.4789	1096.9796	43905.9781	True
5	15	34247.0594	12842.5601	55651.5587	True
7	9	-16721.1571	-38125.6564	4683.3422	False
7	15	-4975.5765	-26380.0758	16428.9228	False
9	15	11745.5806	-9658.9187	33150.0798	False

2-2 comparison (Tukey) – Ammonia condition – *P. stuartii*

Omp-Pst1 porin – FCCs

Multiple Comparison of Means - Tukey HSD,FWER=0.05

	group1	group2	meandiff	lower	upper	reject
4H	0	500	-48214.2397	-94654.66	-1773.8194	True
5H	0	500	-35184.7269	-66101.6521	-4267.8018	True
7H	0	500	-80993.2267	-134113.1314	-27873.3221	True
9H	0	500	-54354.4792	-118532.4498	9823.4914	True
15H	0	500	-63325.4663	-115029.2888	-11621.6438	True

Omp-Pst1 porin – SABs

Multiple Comparison of Means - Tukey HSD,FWER=0.05

	group1	group2	meandiff	lower	upper	reject
7H	0	500	51763.2687	13226.7333	90299.804	True
9H	0	500	44588.3453	-2241.4323	91418.123	False
15H	0	500	122.8988	-69268.6729	69514.4706	False

2-2 comparison (Tukey) – Bicarbonate condition – *P. stuartii*

Omp-Pst1 porin – FCCs

Multiple Comparison of Means - Tukey HSD,FWER=0.05

	group1	group2	meandiff	lower	upper	reject
4h	0	50	-252343.4867	-407839.5819	-96847.3915	True
5h	0	50	-54122.7379	-107260.2379	-985.2379	True
7h	0	50	-53049.4907	-161346.8928	55247.9113	False
9h	0	50	-37971.1624	-256101.1496	180158.8249	False
15h	0	50	5929.5254	-256043.3444	267902.3952	False

Omp-Pst2 porin – FCCs

```
        Multiple Comparison of Means - Tukey HSD,FWER=0.05
        =====================================================
        group1 group2  meandiff    lower      upper    reject
        -----------------------------------------------------
4h        0     50    6895.8395  -59887.8827  73679.5616 False
5h        0     50     345.2546  -17834.8625  18525.3718 False
7h        0     50   11245.2606   -8163.8192  30654.3404 False
9h        0     50      877.444   -9994.3841  11749.2721 False
15h    0     50   16834.8001      595.501  33074.0991  True
```

2-2 comparison (Tukey) – pH condition – *P. stuartii*

Omp-Pst1 porin – FCCs

4h:

```
   Multiple Comparison of Means - Tukey HSD,FWER=0.05
========================================================
group1 group2   meandiff     lower       upper    reject
--------------------------------------------------------
  5      6     30203.4498 -33493.5242  93900.4238 False
  5      7     49864.7744  -2949.9663 102679.5152 False
  5      8     18929.9289 -44767.0451  82626.9029 False
  5      9     -5065.2398 -68762.2138  58631.7342 False
  6      7     19661.3246 -33153.4161  72476.0654 False
  6      8    -11273.5209 -74970.4949  52423.4531 False
  6      9    -35268.6896 -98965.6636  28428.2843 False
  7      8    -30934.8455 -83749.5863  21879.8952 False
  7      9    -54930.0143 -107744.755  -2115.2735  True
  8      9    -23995.1687 -87692.1427  39701.8052 False
```

5h:

```
   Multiple Comparison of Means - Tukey HSD,FWER=0.05
=======================================================
group1 group2   meandiff     lower      upper    reject
-------------------------------------------------------
  5      6     4324.7478  -35825.5557 44475.0512 False
  5      7     19422.9899  -13867.883 52713.8628 False
  5      8     -9858.4211 -50008.7245 30291.8823 False
  5      9    -15480.8529 -55631.1564 24669.4505 False
  6      7     15098.2422 -18192.6308 48389.1151 False
  6      8    -14183.1688 -54333.4722 25967.1346 False
  6      9    -19805.6007 -59955.9041 20344.7027 False
  7      8     -29281.411 -62572.2839 4009.4619  False
  7      9    -34903.8429 -68194.7158 -1612.9699  True
  8      9     -5622.4319 -45772.7353 34527.8716 False
```

7h:

```
    Multiple Comparison of Means - Tukey HSD,FWER=0.05
=========================================================
group1 group2   meandiff     lower        upper    reject
---------------------------------------------------------
  5      6     83222.8583  9312.0111   157133.7054  True
  5      7     77210.3097  15926.6727  138493.9467  True
  5      8     37275.7864 -36635.0608  111186.6335 False
  5      9     6370.9945  -67539.8527   80281.8416 False
  6      7     -6012.5486 -67296.1855   55271.0884 False
  6      8    -45947.0719 -119857.919   27963.7752 False
  6      9    -76851.8638 -150762.7109  -2941.0167  True
  7      8    -39934.5233 -101218.1603  21349.1136 False
  7      9    -70839.3152 -132122.9522  -9555.6783  True
  8      9    -30904.7919 -104815.639   43006.0552 False
```

Omp-Pst1 porin – SABs

7h:

Multiple Comparison of Means - Tukey HSD,FWER=0.05

group1	group2	meandiff	lower	upper	reject
5	6	-26759.8469	-78909.5474	25389.8536	False
5	7	-33941.5292	-76521.5814	8638.5229	False
5	8	-66739.4717	-118889.1722	-14589.7711	True
5	9	-70736.6287	-122886.3292	-18586.9282	True
6	7	-7181.6824	-49761.7345	35398.3698	False
6	8	-39979.6248	-92129.3253	12170.0757	False
6	9	-43976.7818	-96126.4823	8172.9187	False
7	8	-32797.9424	-75377.9946	9782.1098	False
7	9	-36795.0995	-79375.1516	5784.9527	False
8	9	-3997.157	-56146.8576	48152.5435	False

2-2 comparison (Tukey) - Urea condition – *P. stuartii*ΔOmp-Pst2

Omp-Pst1 porin – FCCs

Multiple Comparison of Means - Tukey HSD,FWER=0.05

	group1	group2	meandiff	lower	upper	reject
4h	0	500	-48814.8603	-79244.6239	-18385.0967	True
5h	0	500	-79571.3733	-112223.3454	-46919.4013	True
7h	0	500	-84041.5601	-146780.2218	-21302.8985	True
9h	0	500	-80294.8613	-120605.1033	-39984.6194	True
15h	0	500	-66788.6326	-124584.7873	-8992.4779	True

Omp-Pst1 porin – SABs

Multiple Comparison of Means - Tukey HSD,FWER=0.05

	group1	group2	meandiff	lower	upper	reject
7h	0	500	-51532.3838	-131390.0291	28325.2614	False
9h	0	500	-67561.3871	-143722.56	8599.7859	False
15h	0	500	-85432.9544	-138213.5268	-32652.382	True

2-2 comparison (Tukey) – Ammonia condition – *P. stuartii*ΔOmp-Pst2

Omp-Pst1 porin – FCC

Multiple Comparison of Means - Tukey HSD,FWER=0.05

	group1	group2	meandiff	lower	upper	reject
4h	0	500	-40754.5817	-70288.9453	-11220.2181	True
5h	0	500	-79073.6582	-112314.5001	-45832.8162	True
7h	0	500	-104908.034	-169132.0224	-40684.0456	True
9h	0	500	-108527.7472	-148262.142	-68793.3525	True
15h	0	500	-21559.4458	-79543.416	36424.5245	True

2-2 comparison (Tukey) – pH condition – *P. stuartii*ΔOmp-Pst2

Omp-Pst1 porin – FCC

5h:

Multiple Comparison of Means - Tukey HSD,FWER=0.05

group1	group2	meandiff	lower	upper	reject
5	6	17463.0878	-29975.2601	64901.4357	False
5	7	61544.038	22810.7891	100277.2868	True
5	8	31719.0228	-15719.3251	79157.3706	False
5	9	11726.8916	-35711.4563	59165.2394	False
6	7	44080.9502	5347.7013	82814.199	True
6	8	14255.935	-33182.4129	61694.2828	False
6	9	-5736.1962	-53174.5441	41702.1516	False
7	8	-29825.0152	-68558.2641	8908.2336	False

7	9	-49817.1464	-88550.3953	-11083.8976	True
8	9	-19992.1312	-67430.4791	27446.2167	False

7h:

Multiple Comparison of Means - Tukey HSD,FWER=0.05

group1	group2	meandiff	lower	upper	reject
5	6	25669.2245	-61218.4629	112556.9119	False
5	7	82700.2695	11756.7698	153643.7692	True
5	8	12770.8041	-74116.8833	99658.4916	False
5	9	-14547.9893	-101435.6767	72339.6981	False
6	7	57031.045	-13912.4547	127974.5447	False
6	8	-12898.4204	-99786.1078	73989.267	False
6	9	-40217.2138	-127104.9013	46670.4736	False
7	8	-69929.4654	-140872.9651	1014.0343	False
7	9	-97248.2588	-168191.7585	-26304.7591	True
8	9	-27318.7935	-114206.4809	59568.894	False

9h:

Multiple Comparison of Means - Tukey HSD,FWER=0.05

group1	group2	meandiff	lower	upper	reject
5	6	21948.695	-38626.9602	82524.3501	False
5	7	84516.5196	35056.7043	133976.3349	True
5	8	43319.6633	-17255.9918	103895.3184	False
5	9	-22639.6063	-83215.2615	37936.0488	False
6	7	62567.8246	13108.0093	112027.6399	True
6	8	21370.9683	-39204.6868	81946.6235	False
6	9	-44588.3013	-105163.9564	15987.3538	False
7	8	-41196.8563	-90656.6716	8262.959	False
7	9	-107156.1259	-156615.9412	-57696.3107	True
8	9	-65959.2696	-126534.9248	-5383.6145	True

Supplementary Table S3 – Statistical analysis from DLS experiments on porin propensity to self-associate in DOTs. All experiments were conducted for at least three biologically independent replicates. Technical replicates were averaged to produce replicate means that were subsequently used for analysis. Mean values were compared within and between groups using one-way ANOVA followed by Tukey's post hoc for two-two comparisons. Differences were considered statistically significant if $p<0.05$ (True).

GLM - DLS

Omp-Pst1 porin – All statistic parameters:

```
                 Generalized Linear Model Regression Results
==============================================================================
Dep. Variable:                      Y   No. Observations:                  389
Model:                            GLM   Df Residuals:                      381
Model Family:                   Gamma   Df Model:                            7
Link Function:          inverse_power   Scale:                        0.097908
Method:                          IRLS   Log-Likelihood:                -1972.8
Date:                Mon, 18 Mar 2019   Deviance:                       33.696
Time:                        14:17:24   Pearson chi2:                     37.3
No. Iterations:                     7   Covariance Type:             nonrobust
===================================================================================
                      coef    std err          z      P>|z|      [0.025      0.975]
-----------------------------------------------------------------------------------
Intercept           0.0137      0.002      8.950      0.000       0.011       0.017
Cporin             -0.0130      0.002     -7.730      0.000      -0.016      -0.010
Urea               2.3e-07   6.93e-07      0.332      0.740   -1.13e-06    1.59e-06
Cporin:Urea      7.344e-07   8.34e-07      0.881      0.378      -9e-07    2.37e-06
Ammonium         3.751e-06   9.29e-07      4.036      0.000    1.93e-06    5.57e-06
Cporin:Ammonium   4.42e-06   1.36e-06      3.246      0.001    1.75e-06    7.09e-06
pH                 -0.0007      0.000     -3.151      0.002      -0.001      -0.000
Cporin:pH           0.0012      0.000      5.136      0.000       0.001       0.002
===================================================================================
```

Omp-Pst2 porin – All statistic parameters:

```
                 Generalized Linear Model Regression Results
==============================================================================
Dep. Variable:                      Y   No. Observations:                  386
Model:                            GLM   Df Residuals:                      378
Model Family:                   Gamma   Df Model:                            7
Link Function:          inverse_power   Scale:                         0.36485
Method:                          IRLS   Log-Likelihood:                -2501.6
Date:                Mon, 18 Mar 2019   Deviance:                       123.99
Time:                        14:19:20   Pearson chi2:                     138.
No. Iterations:                     8   Covariance Type:             nonrobust
===================================================================================
                      coef    std err          z      P>|z|      [0.025      0.975]
-----------------------------------------------------------------------------------
Intercept           0.0066      0.001      4.551      0.000       0.004       0.010
Cporin             -0.0046      0.001     -3.105      0.002      -0.008      -0.002
Urea             4.958e-07   6.87e-07      0.722      0.470   -8.51e-07    1.84e-06
Cporin:Urea     -6.935e-07   6.79e-07     -1.022      0.307   -2.02e-06    6.36e-07
Ammonium         1.284e-05   1.92e-06      6.681      0.000    9.07e-06    1.66e-05
Cporin:Ammonium -1.208e-05   1.82e-06     -6.651      0.000   -1.56e-05   -8.52e-06
pH                 -0.0003      0.000     -1.475      0.140      -0.001    9.77e-05
Cporin:pH           0.0002      0.000      1.107      0.268      -0.000       0.001
===================================================================================
```

Omp-Pst1 porin – Bicarbonate – All statistic parameters:

```
                 Generalized Linear Model Regression Results
==============================================================================
Dep. Variable:                      Y   No. Observations:                   96
Model:                            GLM   Df Residuals:                       92
Model Family:                   Gamma   Df Model:                            3
Link Function:          inverse_power   Scale:                       0.0066722
Method:                          IRLS   Log-Likelihood:                -380.86
Date:                Sat, 20 Apr 2019   Deviance:                      0.62057
Time:                        11:22:54   Pearson chi2:                    0.614
No. Iterations:                     7   Covariance Type:             nonrobust
=============================================================================================
                                coef    std err          z      P>|z|      [0.025      0.975]
---------------------------------------------------------------------------------------------
Intercept                     0.0129      0.000     60.130      0.000       0.012       0.013
Bicarbonate                2.458e-06   4.09e-06      0.601      0.548   -5.56e-06    1.05e-05
Concentration                -0.0109      0.000    -50.047      0.000      -0.011      -0.010
```

```
Bicarbonate:Concentration  1.985e-05   4.23e-06      4.691      0.000    1.16e-05    2.81e-05
=============================================================================================
```

Omp-Pst1 porin – Bicarbonate:

```
                 Generalized Linear Model Regression Results
==============================================================================
Dep. Variable:                      Y   No. Observations:                   96
Model:                            GLM   Df Residuals:                       94
Model Family:                   Gamma   Df Model:                            1
Link Function:          inverse_power   Scale:                         0.49076
Method:                          IRLS   Log-Likelihood:                -601.92
Date:                Sat, 20 Apr 2019   Deviance:                       56.351
Time:                        11:23:18   Pearson chi2:                     46.1
No. Iterations:                     7   Covariance Type:             nonrobust
===============================================================================
                  coef    std err          z      P>|z|      [0.025      0.975]
-------------------------------------------------------------------------------
Intercept       0.0037      0.000      8.701      0.000       0.003       0.005
Bicarbonate   2.78e-05   1.06e-05      2.634      0.008    7.11e-06    4.85e-05
===============================================================================
```

Omp-Pst2 porin – Bicarbonate – All statistic parameters:

```
                 Generalized Linear Model Regression Results
==============================================================================
Dep. Variable:                      Y   No. Observations:                   50
Model:                            GLM   Df Residuals:                       48
Model Family:                   Gamma   Df Model:                            1
Link Function:          inverse_power   Scale:                        0.016299
Method:                          IRLS   Log-Likelihood:                -246.36
Date:                Sat, 20 Apr 2019   Deviance:                      0.76802
Time:                        11:34:22   Pearson chi2:                    0.782
No. Iterations:                    18   Covariance Type:             nonrobust
=============================================================================================
                                coef    std err          z      P>|z|      [0.025      0.975]
---------------------------------------------------------------------------------------------
Intercept                     0.0013   3.89e-05     33.427      0.000       0.001       0.001
Bicarbonate                1.703e-05   1.17e-06     14.569      0.000    1.47e-05    1.93e-05
Concentration                 0.0013   3.89e-05     33.427      0.000       0.001       0.001
Bicarbonate:Concentration  1.703e-05   1.17e-06     14.569      0.000    1.47e-05    1.93e-05
=============================================================================================
```

Omp-Pst2 porin – Bicarbonate:

```
                 Generalized Linear Model Regression Results
==============================================================================
Dep. Variable:                      Y   No. Observations:                   50
Model:                            GLM   Df Residuals:                       48
Model Family:                   Gamma   Df Model:                            1
Link Function:          inverse_power   Scale:                        0.016299
Method:                          IRLS   Log-Likelihood:                -246.36
Date:                Sat, 20 Apr 2019   Deviance:                      0.76802
Time:                        11:35:15   Pearson chi2:                    0.782
No. Iterations:                     6   Covariance Type:             nonrobust
===============================================================================
                  coef    std err          z      P>|z|      [0.025      0.975]
-------------------------------------------------------------------------------
Intercept       0.0026   7.79e-05     33.427      0.000       0.002       0.003
Bicarbonate  3.405e-05   2.34e-06     14.569      0.000    2.95e-05    3.86e-05
===============================================================================
```

2-2 comparison (Tukey) – Ammonia condition

Omp-Pst1 porin

```
Multiple Comparison of Means - Tukey HSD,FWER=0.05
=================================================
group1 group2 meandiff   lower     upper   reject
-------------------------------------------------
  0     100   -226.57  -347.8389 -105.3011  True
  0     500   -207.49  -328.7589  -86.2211  True
  0     1000  -230.26  -351.5289 -108.9911  True
 100    500    19.08   -120.9493  159.1093 False
 100    1000   -3.69   -143.7193  136.3393 False
```

```
 500    1000   -22.77  -162.7993  117.2593 False
```

Omp-Pst2 porin

```
Multiple Comparison of Means - Tukey HSD,FWER=0.05
===============================================
group1 group2 meandiff  lower    upper   reject
-----------------------------------------------
  0     100    98.33     2.99    193.67   True
  0     500    193.62   98.28    288.96   True
  0     1000   308.39  205.411  411.369   True
 100    500    95.29   -14.7991 205.3791 False
 100    1000   210.06  93.2928  326.8272  True
 500    1000   114.77  -1.9972  231.5372 False
```

2-2 comparison (Tukey) – Bicarbonate condition

Omp-Pst1 porin

```
Multiple Comparison of Means - Tukey HSD,FWER=0.05
=================================================
group1 group2 meandiff   lower     upper   reject
-------------------------------------------------
  0      10    -57.92  -125.4145   9.5745  False
  0      25    -71.63   -126.739  -16.521   True
  0      50    -185.4   -240.509  -130.291  True
  0     100   -260.72   -315.829  -205.611  True
  10     25    -13.71   -81.2045  53.7845  False
  10     50   -127.48  -194.9745  -59.9855  True
  10    100    -202.8  -270.2945 -135.3055  True
  25     50   -113.77   -168.879  -58.661   True
  25    100   -189.09   -244.199  -133.981  True
  50    100    -75.32   -130.429  -20.211   True
```

Omp-Pst2 porin

```
Multiple Comparison of Means - Tukey HSD,FWER=0.05
=================================================
group1 group2 meandiff   lower     upper   reject
-------------------------------------------------
  0      10   -116.89  -151.1648  -82.6152  True
  0      25   -139.98  -174.2548 -105.7052  True
  0      50   -221.96  -256.2348 -187.6852  True
  0     100   -242.87  -277.1448 -208.5952  True
  10     25    -23.09   -57.3648  11.1848  False
  10     50   -105.07  -139.3448  -70.7952  True
  10    100   -125.98  -160.2548  -91.7052  True
  25     50    -81.98  -116.2548  -47.7052  True
  25    100   -102.89  -137.1648  -68.6152  True
  50    100    -20.91   -55.1848  13.3648   True
```

2-2 comparison (Tukey) – pH condition

Omp-Pst1 porin

```
Multiple Comparison of Means - Tukey HSD,FWER=0.05
=================================================
group1 group2 meandiff   lower     upper   reject
-------------------------------------------------
  5      6     -110.6  -137.2876  -83.9124  True
  5      7     14.73    -11.9576  41.4176  False
  5      8    -192.42  -219.1076 -165.7324  True
  5      9    -165.36  -192.0476 -138.6724  True
  6      7     125.33   98.6424   152.0176  True
  6      8     -81.82  -108.5076  -55.1324  True
  6      9     -54.76   -81.4476  -28.0724  True
  7      8    -207.15  -233.8376 -180.4624  True
  7      9    -180.09  -206.7776 -153.4024  True
  8      9     27.06     0.3724   53.7476   True
```
